# Breeding and milking managements and Goat production constraints in Siltie Zone SNNPR, Ethiopia

**DOI:** 10.1016/j.heliyon.2023.e22573

**Published:** 2023-11-22

**Authors:** Ahmed Hussein, Serkalem Aseffa, Belete Kuraz, Bushra Bedaso

**Affiliations:** Department of Animal Science, Werabe University, P.O.Box.46, Werabe, Ethiopia

**Keywords:** Production constraints, Animal selection

## Abstract

The study was conducted in three agroecology of the Siltie Zone and assessed Breeding practice, Milking practice, and Goat Production Constraints in Siltie Zone SNNPR, Ethiopia. Multistage sampling techniques were followed during determining the study area where finally 384 respondents (128 from each agroecology) were selected to administer questionnaires. Primary data was collected through field observation, interviews, pretested questionnaires, and discussion with Focus Group Discussion. Collected data was organized into Microsoft Excel 2010 and analyzed using SPSS 21. The current study indicated respondents reared goats for multiple purposes, among which income generation (index = 0.38), followed by meat and breeding (index = 0.24) and milk (index = 0.19) ranked 1st, 2nd^,^ and 3rd respectively. Phenotypically breeding bucks were selected primarily based on large body size, Body confirmation, and coat color whereas breeding does were selected based on Udder size, milk yield, litter size, and body confirmation. The current study shows that the Majority (59.90 %) of the bucks utilized for breeding purposes originated from respondents' flocks while government and neighbor-sourced contribute 22.40 % and 18.49 % respectively. Lowland goats were comparably potential milk yielders (0.655 ± 0.18) and larger in litter size (LS). As understood from this study, Feed shortage (index = 0.14), water shortage (index = 0.11), and land shortage (index = 0.10) were dry season and feed shortage (index = 0.26), land shortage (index = 0.19) and Productive Breeds (index = 0.07) were rainy season constraints distressing goat production in Siltie Zone. The current study points out that farmers should be aware of flock separation practice and its importance and supported by a Selection-based breeding program to handle only productive buck to improve goat productivity and reproductive performance. Furthermore facilitating access to improved forage, using hay and crop residue were options suggested to overcome feed shortage.

## 1 Introduction

Ethiopia is first in Africa and fifth largest in the world by livestock potential with its increasing rate [1]. Currently, the small ruminant populations are 42.9 million sheep and 52.2 million goats in the country [2] and nearly all goats (99.99 %) are indigenous breeds to Ethiopia [3,4] Small ruminants, particularly native breeds, play a significant role in the livelihoods of a considerable part of the human population in the tropics from socio-economic aspects [[5], [6], [7]].

Goats are among the most preferred livestock species [8] and are kept for multiple purposes of improving smallholder livelihoods [8,9]. Goats are a primary income source [10] that serves as a source of cash income to pay for school fees for children, and the purchase of goods such as clothes, salt, and sugar for the family [11]. Additionally, it serves as household security, accumulating capital, and fulfilling cultural obligations [[12], [13], [14], [15]] source of other valuable non-food products such as skin and manure [16]. Furthermore, they could be used for immediate cash sources, milk, meat, wool, manure, and saving or risk distribution [16,17].

The national herd supports the livelihoods of more than 11.3 million rural households, including 27–35 % of the highland livestock keepers, and a large proportion of the lowland herders, who live below the poverty line [18]. The diverse agroecological zone of a country makes it suitable to support large numbers and breeds of goats [19] where lowlands maintain about three-quarters of the goat population [20]. Goats contribute substantially to the livelihood and income of the rural and the country at large [21] due to their adaption to various agro-ecologies [22], low cost of production, dynamic feeding behavior, high fertility and growth rates and fast reproduction cycle goats are preferably reared by various ethnic communities and found in all production systems and rural low-income farmers for a different purpose [23].

Thus, combined trials with emphasis on administration and breeding to improve animal outputs are of decisive significance [24,25]. The economic and biological efficiency of animal production enterprises generally improved by increasing the productivity and reproductive performance of animals [26,27]. In all Agroecology of Ethiopia farmers’ selection practice helps to select the goat based on economic traits that contribute to economic value [28]. Selection criteria may vary based on agroecology and the sex of the goats. In agro-pastoral goat production systems, milk yield, fast growth, body size, and weaning and twining ability were the most highly rated goat traits [29]. A study made by Ref. [20] confirmed that body conformation, coat color, twining ability, and adaptation were the most preferred goat traits in the lowland. As [30] reported, traits like body size, body conformation (height, length, and appearance), and coat color are important traits given due emphasis in selecting breeding bucks. Different [[31], [32], [33]], also reported that body size; twinning ability (multiple births), and milk yield are listed as preferred traits to select breeding.

Reproductive performances of the goats are usually measured through traits such as age at puberty, age at first kidding, kidding interval, and litter size [34] which is important for breed improvement under a given agro system. According to Ref. [35] report the age at puberty of the local breed for bucks and doe reared in the Amharic Region were 8–10 and 6–8 months respectively [36]. Reported the averages of kidding interval and age at first kidding intervals of the local breed of goats (breeds not identified) were 11.52 ± 0.96 months and 13.18 ± 1.45 months respectively in the Adaa Liben districts of East Showa, Ethiopia.

The milk yield of the native goats is low as most of them are not of true dairy-type breeds, indicating that the average lactation milk length and average daily milk yields of goats reared in Ethiopia are 3 months and 351.28g/days respectively however problems associated with drought, disease, feed shortages, predator and market problems aggravate the problems further [35].

Despite the large number, of their contribution to support the livelihood of the farmers and the national economy, their productivity in Ethiopia is low [22]. Different scholars ([37,38] reported that the major constraints of goat production in Ethiopia are infectious and parasitic diseases, feed shortage, lack of superior genotype in order, management, high predatory, and market instability and water shortage. Furthermore, scarcity and untimely credit access were the major constraints that hindered goat productivity [39].

Prior to conducting study on goat production and productivity improvement information obtained from the siltie zone [40] showed that there is a need for study as previously limited investigations were performed in the selected study areas. So The present study was aimed at evaluating farmer-level animal selection practices for breeding, milking practices, reproductive performance, and constraints of goats' production under three Ethiopian agroecological siltie zone.

## 2 Materials and methods

### 2.1 Description of study area

The study was conducted in a Silt zone having a total area of 3000 sq. km and situated 173 km from Addis Ababa. This zone is bordered in North West by the Alaba Zone, North East by the Hadiya Zone, West by Oromia, and South, South East, and South West by the Gurage Zone. The human population of the Zone is 831,573 of which 51 % are male and 49 % female. It is classified into three agroecology where 20.6 % is highland (2300–3200 m), 74.4 % is midland (1500–2300), and 5 % is lowland (500–1500)m. The mean annual temperature ranges between 12 °C and 26 °C. The rainfall is between 700 and 1818 mm. Agriculture is the main economic activity [41].

### 2.2 Sampling techniques and sample size determination

Multi-stage sampling techniques were followed to select the study area and sample size. Potential districts were determined based on information obtained from the Siltie Zone livestock and fishery department [40]SZLFD, 2019) and then one district was randomly selected from each agroecology (Fig. 3). Secondly, PA (peasant Association) with more populated goat numbers were determined at the district level, and the highest two were selected from each. Farmers owned more than six goats (n > 6) and their prior experience of goat rearing was considered as selection criteria. In consultation with the Development Agent, 384 respondents (128 Households from one agroecology) were selected by simple random sampling and then administered questionnaires. A sample size was calculated using [42].

n = N.

1 + N (e) ^2^

Where = n Number of Sample to be taken

N = Total Population of Study Area (population size)

e^2^ = random error of a sample.

N = 831,573, e = 0.07

n = 831,573 = 384,

1 + 831,573 (0.05)2

### 2.3 Data collection

The study encompasses both primary and secondary sources of data. Primary data was collected through field observations and semi-structured questionnaires. The questionnaires were prepared to get information on selection practices, the purpose of goat handling, labor management, and reproductive performance traits such as age at first mating, age at first kidding, kidding interval, and litter size (prolificacy) of goats. Secondary data was collected from written documents from the Siltie Zone livestock and fishery department, specific study areas different journals, books, and published articles.

### 2.4 Data analysis

The data collected were organized by their order of collection and compiled in Microsoft Excel 2010 then analyzed using [43] software version 21. The index was calculated to provide the Rank on feed resources and Factors affecting goat production. A non-parametric test like the Chi-square test was used to explain the difference that may exist in qualitative parameters. Index = Σ [nrank1+n-1 (for rank (1 + 1) + n-2for rank (1 + 2) + n-3 (for rank1+3 … ….] given for an individual reason divided by Σ[nrank1+n-1 (for rank (1 + 1) + n-2for rank (1 + 2) + n-3 (for rank1+3 … ….] [44].

## 3 Results

### 3.1 Purpose of keeping goats

The average index of the Current study indicated that goats serve farmers with multiple roles, primarily as income generation (index = 0.38), followed by meat and breeding (index = 0.24) and milk (index = 0.19) ranked 1st, 2nd^,^ and 3rd respectively (Table 1). Goat handling purpose Variation was observed among agroecology where lowland respondents handled Goats primarily for milk followed by income, and breeding while midland and highland farmers handled Goats primarily for income, meat, and breeding ranked 1st, 2nd, and 3rd respectively.

**Table 1:** Purposes of keeping goats handling.

Purpose of goat rearing	Lowland		Midland		Highland		Overall	
	Index	Rank	Index	Rank	Index	Rank	Index	Rank
Meat	0.15	4	0.23	2	0.33	2	0.24	2
Milk	0.37	1	0.14	4	0.05	5	0.19	3
Income	0.31	2	0.36	1	0.46	1	0.38	1
Breeding	0.23	3	0.19	3	0.29	3	0.24	2
Savings	0.09	5	0.12	5	0.23	4	0.15	5

Index = (R1*5 + R2*4 + R3*3 + R4*2 + R5*1) for a variable divided by/Sum of (R1*5 + R2*4 + R3*3 + R4*2 + R5*1) for all variables.

### 3.2 Labor management activity

The current study shows that all member of the household has a valuable contribution in rearing goat even though there were role differences observed among gender (Table 2). Males were mostly allowed to focus on outdoor activities while females were limited to indoor activities traditionally conceptualized as ability differences. There is statistical variation between agro-ecology (P < 0.05) in the herding of large ruminants were majorly by Father at lowland (54.57 %) and highland (60.34 %) whereas by Hired labor (46.25 %) at midland. The overall results of the current study show that large ruminant herding was performed by the son (25.82 %) and father (48.22 %). Small ruminant herding variation (P < 0.05) was observed across agro-ecology where Father (48.36 %), Hired labor (53.19 %), and Son (42.69 %) contributed higher at lowland, midland, and highland respectively. Furthermore milking goats, cleaning of house and barn, and selling animal products were performed by the mother (52.97 %, 52.29 %, and 70.11 %) and daughter (42.66 %, 27.92 %, and 27.71 %) respectively.

**Table 2:** Labor management for different livestock-related activities under study area.

Activities	Agroecology	Sons (%)	Daughters (%)	Father (%)	Mother (%)	Hired labor (%)	P Value
Herding of large ruminants (cow, ox, heifer, and bull)	Lowland	31.35	3.48	54.57	10.6		**
	Midland	15.61		29.74	8.4	46.25	
	Highland	30.5		60.34		9.16	
	Overall	25.82	1.16	48.22	6.33	18.47	
Herding of small ruminants (sheep and goats)	Lowland	38.12	13.52	48.36			*
	Midland	3.9	17.63	25.28		53.19	
	Highland	42.69	39.47	17.84			
	Overall	28.24	23.54	30.49		17.73	
Caring for young, sick animals and	Lowland	6.32	37.16		52.23	4.29	ns
	Midland	6.98	38.62	8.13	46.27		
	Highland	10.62	41.18	2	46.2		
	Overall	7.97	38.99	3.38	48.23	1.43	
Milking animals (goats)	Lowland	2.8	39.82		57.38		ns
	Midland	1.11	47.22		51.67		
	Highland		40.93	9.2	49.87		
	Overall	1.30	42.66	3.07	52.97		
Cleaning of house and barn	Lowland	11.20	36.82	4.88	47.10		*
	Midland		24.87		31.84	43.29	
	Highland		22.08		77.92		
	Overall	3.73	27.92	1.63	52.29	14.43	
Buying and selling of live animals	Lowland	18.38		81.62	14.6		*
	Midland	26.19		73.81			
	Highland	12.4		47.7	39.9		
	Overall	18.99		67.71	18.17		
Selling of animal products (milk, cheese, butter, and yogurts)	Lowland		23.51		76.49		ns
	Midland		17.5		82.5		
	Highland		42.13	6.53	51.34		
	Overall		27.71	2.18	70.11		
Building a human house and livestock shelter	Lowland	33.75	8.35	47.9	10.35		**
	Midland	16.43		41.27		42.3	
	Highland	5		40.19		49.81	
	Overall	18.39	2.78	43.12	3.45	30.70	

% = percent of respondents, * = Significant at P < 0.05, ** = Significant at P < 0.001, ns = not significant.

### 3.3 Breeding practices

Overall data from Table 3 show that 59.90 % of respondents depend on breeding bucks originating from their flocks. None of the respondents (P > 0.05) use buck sourced from the government at the highland nevertheless, 39.06 % and 28.13 % of households in midland and lowland. Out of 384 respondents, 70.31 % replayed as they implemented flock separation majorly based on age (54.95 %) and condition (37.50 %).

**Table 3:** Breeding practices of the goat in the study area.

Variables		Lowland	Midland	Highland	Overall	P value
		N (%)	N (%)	N (%)	N (%)	
Breeding Buck Source	Own flock	80 (62.50)	63 (49.22)	87 (67.97)	230 (59.90)	**
	Neighbors	12 (9.37)	15 (11.72)	44 (34.38)	71 (18.49)	
	Government	36 (28.13)	50 (39.06)	0.00	86 (22.40)	
Do You, Separation Flock	Yes	96 (75.00)	83 (64.84)	91 (71.09)	270 (70.31)	ns
	No	32 (25.00)	45 (35.16)	37 (28.91)	114 (29.69)	
The basis for flock separation	Ages	81 (63.28)	46 (35.94)	84 (65.63)	211 (54.95)	**
	By Sex	9 (7.03)	12 (9.38)	8 (6.25)	29 (7.55)	
	Condition	38 (29.69)	70 (54.69)	36 (28.13)	114 (37.50)	
know and prevent the Inbreeding problem	Yes	39 (30.47)	63 (49.22)	48 (37.50)	150 (39.06)	ns
	No	72 (56.25)	65 (50.78)	80 (62.50)	217 (56.51)	

Source own survey, 2019, N = number of respondents, % = percent of respondents from the study compared to total respondents, ** = Significant at P < 0.001, ns = not significant.

### 3.4 Milk yield and lactation length

Lactation length Shaw similarity (P > 0.05) at highland and midland agroecology. There was a significant difference (P < 0.05) in daily milk yield per day of goats across agro-ecology where the higher result was reported from lowland (0.655 ± 0.18) followed by midland (0.37 ± 0.11) and highland (0.29 ± 0.19). The mean value of the daily milk yield of goats was reported 0.44 ± 0.07 L/day (Table 4)

**Table 4:** Average percentage of Productive performance of goats in the study area.

Variables	Lowland	Midland	Highland	Overall	P *value*
	Mean ± SE	Mean ± SE	Mean ± SE	Mean ± SE	
Milk yield (Litter)	0.65 ± 0.18^a^	0.37 ± 0.11^b^	0.29 ± 0.19^c^	0.44 ± 0.16	*
Lactation length (Month)	3.25 ± 0.21^a^	2.9 ± 0.34^b^	2.89 ± 0.09^b^	3.01 ± 0.21	*

a-b different superscript letters within the same row: P < 0.05, N = number of respondents, % = percent of respondents, * = significant at P < 0.05.

### 3.5 Milking practice of Goat at study area

The current study shows that the purpose of milking varies (P < 0.05) among agroecology. Lowland respondents milk goats majorly (85.16 %) for home consumption however 56.25 % of midland and 75.78 % of highland respondents do not milk goats (Table 5). The overall percentage indicated that the goat was milked mostly (70.57 %) by mothers and given to children (80.21 %). Plastic material was used by 77.86 % of respondents as milking equipment. Most midland and highland respondents clean milking equipment once per day; however, lowland respondents clean twice per day. Water and soap were more utilized cleaning materials in midland (83 %) and highland (78.91 %)but water followed by fumigation was more practiced in lowland.

**Table 5:** Milking practice at study area.

Activities		Lowland		Midland		Highland		Overall		P value
		N	%	N	%	N	%	N	%	
Purpose of milking	Home consumption	109	85.16	56	43.75	31	24.22	196	51.04	*
	Income (sale)	9	7.03	0	0.00	0	0.00	9	2.34	
	Not milked	10	7.81	72	56.25	97	75.78	179	46.61	
Milker	Mother	87	67.97	74	57.81	110	85.94	271	70.57	ns
	Young female	41	32.03	54	42.19	18	14.06	113	29.43	
Milk utilizers	Children	102	79.69	91	71.09	115	89.84	308	80.21	ns
	Elders	20	15.63	35	27.34	0	0.00	55	14.32	
	Women	6	4.69	2	1.56	0	0.00	8	2.08	
Milking equipment	Plastic	99	77.34	84	65.63	116	90.63	299	77.86	ns
	Metallic	10	7.81	0	0.00	0	0.00	10	2.60	
	Traditional	19	14.84	32	25.00	12	9.38	63	16.41	
Frequency of cleaning	Once per day	43	33.59	100	78.13	128	100.00	271	70.57	*
	Twice per day	85	66.41	28	21.88	0	0.00	113	29.43	
Milk cleaning material	Water and soap	26	20.31	83	64.84	101	78.91	210	54.69	*
	Water and fumigation	91	71.09	45	35.16	15	11.72	151	39.32	
	Water, soap, and fumigation	11	8.59	0	0.00	12	9.38	51	5.99	

* = Significant at P < 0.05, N = number of respondents, % = percent of respondents, ns = not significant at P < 0.05.

### 3.6 Reproductive performance of goat

The results pertaining to the reproductive performance of the goats reared in the study area was presented in Table 6. The average AFM, AFS, AFK, and LS of goats were 7.64 ± 0.58, 7.82 ± 0.764, 14.00 ± 0.93 and 1.59 ± 0.28 respectively. There were differences in the reproductive performance of goats across agro-ecology where Longer AFM (male), AFS (female), and AFK were reported from the lowland of the siltie zone while KI doesn't Shaw variation (P > 0.05) across agroecology. A larger litter size (P < 0.05) was reported from the lowland (1.945 ± 0.18).

**Table 6:** Mean (±) SEM Reproductive Performance of goats from the study area.

Reproductive Variables	Lowland	Midland	Highland	Overall	P value
AFM (month)	8.21 ± 0.7^a^	7.685 ± 0.29^b^	7.035 ± .0.73^c^	7.64 ± 0.58	**
AFS (month)	8.18 ± 0.17^a^	7.49 ± 0.47^c^	7.78 ± 0.47^b^	7.82 ± 0.764	*
AFK (month)	14.49 ± 0.66^a^	14.05 ± 0.57^b^	14.00 ± 0.62^b^	14.00 ± 0.93	*
KI (month)	6.31 ± 0.36	6.56 ± 0.34	6.23 ± 0.37	6.70 ± 0.53	ns
LS (number)	1.95 ± 0.18^a^	1.43 ± 0.18^b^	1.39 ± 0.19^b^	1.59 ± 0.28	**

a-c different superscript letters within the same row: P < 0.05, * = Significant at P < 0.05, ** = Significant at P < 0.001, N = number of respondents, SEM = standard error of the mean, P < 0.05, AFM = Age at first mating, AFS= Age at first service, AFK= Age at first kidding, KI= Kidding interval, LS= Litter Size.

### 3.7 Selection of breeding buck and doe

Respondent's Goat selection practice of bath sex (Buck and Due) for the breeding purpose was presented in Table 7. This study specified that respondents' goat selection criteria and practice vary with sex and agroecology. Lowland respondents select breeding buck primarily by body conformation (index = 0.31), large body size (index = 0.21), presence of horn (index = 0.16), and behavior (index = 0.11) while breeding doe based on body conformation (index = 0.40), udder size (index = 0.27), milk yield (index = 0.17) and litter size (index = 0.13) ranked 1st, 2nd, 3rd, and 4th respectively. At midland and highland, Breeding buck was selected via body size, coat color, and presence of horn. Breeding doe was selected via litter size, coat color, and large body at midland via large body size, litter size, and coat color at highland ranked 1st, 2nd, and 3rd respectively.

**Table 7:** Index and rank Goat selection criteria and practices in the study area.

Goat selection criteria	Lowland				Midland				Highland			
	Male		Female		Male		Female		Male		Female	
	Index	Rank	Index	Rank	Index	Rank	Index	Rank	Index	Rank	Index	Rank
Coat color	0.06	5	0.05	8	0.39	2	0.24	2	0.13	3	0.15	3
Behavior	0.11	4	0.09	7	0.08	6			0.10	5		
Large Body size	0.21	2			0.39	1	0.16	3	0.16	1	0.17	1
Presence of horns	0.16	3	0.09	5	0.30	3	0.10	8	0.14	2	0.15	4
Udder sizes			0.27	2			0.11	6			0.12	6
Litter sizes			0.13	4			0.29	1			0.17	2
Body conformation	0.31	1	0.40	1	0.13	4	0.11	5	0.13	3	0.06	8
High Milk yield			0.17	3			0.11	6			0.14	5
Disease tolerance	0.04	6	0.11	6	0.12	5	0.15	4			0.12	7

* = not selected (not indexed and ranked), Index= (R1 * 7 + R2*6 + R3*5 + R4*4 + R5*3 + R6*2 + R7*1) for a variable divided by/Sum of (R1 * 7 + R2*6 + R3*5 + R4*4 + R5*3 + R6*2 + R7*1) for all variables, indices value range up to 8 for female goat and up to 6 for males.

### 3.8 Constraints of Goat production in study area

Different goat production constraints that hamper goat production in the study area were listed in Table 8. The overall index revealed that Feed shortage (index = 0.14), water shortage (index = 0.11), and land shortage (index = 0.10) were major goat production constraints during the dry season. There were differences in constraint types across agroecology and season where, drought, water, and feed shortage at lowland; feed, water, and disease at midland, and; feed and land shortage at highland ranked 1st, 2nd^,^ and 3rd during the dry season. Feed shortage was a common primary goat production constraint in both season and agroecology.

**Table 8:** Index of goat production Constraints of Goat production in the study area.

Variables	Lowland		Midland		Highland		Overall			
	Season		Season		Season		Season			
	Dry	Rainy	Dry	Rainy	Dry	Rainy	Dry	Rank	Rainy	Rank
Drought Problem	0.27	*	0.02	*	*	*	0.09	4	*	
Water Shortage	0.16	*	0.13	*	*	*	0.11	2	*	
Feed Shortage	0.11	0.36	0.14	0.12	0.19	0.31	0.14	1	0.26	1
Land Shortage	*	0.25	*	0.14	0.29	0.19	0.10	3	0.19	2
Disease and Parasite	*	0.09	0.10	0.10	*	*	0.03	7	0.06	4
Health Service	0.07	0.06	*	*	0.09	0.03	0.05	6	0.03	5
Productive Breeds	0.03	0.03	0.06	0.09	0.12	0.08	0.07	5	0.07	3

Index= (R1 * 5 + R2*4 + R3*3 + R4*2 + R5*1) for a variable divided by/Sum of (R1 * 5 + R2*4 + R3*3 + R4*2 + R5*1) for all variables, * = none of respondent selected the level.

## 4 Discussion

### 4.1 Purposes of keeping goats

In the study area respondents handle goats for multiple purposes primarily for income (index = 0.38) followed by meat (index = 0.24) and milk (index = 0.19). Similarly study conducted by Refs. [7,8] reported that Goats are kept for multiple purposes of improving smallholder livelihoods. Goat keeping primary for income generation reported in the current study was consistent with the result reported by Ref. [45] who made an investigation on the Benishangul Gumuz region and [46] who made an investigation on East Arsi Zone, Oromia region. Focus group discussions conducted with respondents revealed that goats were used as income generation due to their fast growth rate, ease of sale, and replacement under any circumstance compared to large Ruminants and used to cover various expenses (Fig. 4). Similar to this finding [47] reported that small ruminants are easily sold when compared with bovines [30,48]. stated that cash income from the sale of goats was spent on purchasing farm inputs, school fees, and re-stocking. The goat keeping for milk followed by income generation purpose reported from the lowland was in line with [38,46,49].

### 4.2 Labor management activity

This study showed all members of the household have valuable contribution in rearing goat even though role differences among gender was experienced. According to FGD made with selected respondents (Fig. 6) Male members of the household were allowed to control most of the outdoor activities like herding of small and large ruminants, selling and purchasing of animals, and building of house and shelter, whereas female members of the family were allowed to perform activities in and around the home like cleaning house and barn, rearing of the young and sick animal, milking of animals and selling of milk and milk by-product at the market). In agreement with the current result [50], reported that, large ruminant herding practices and management fall under the control of men [51]. reported that the daily removal of home wastes and dung from the barn/shade and care of sick animals were categorized as women's roles.

### 4.3 Breeding practices

The majority (59.90 %) of the breeding bucks used for the breeding purpose originated from respondents' flocks. None of the respondents (P > 0.05) use bucks sourced by the government at the highland. However, 39.06 % and 28.13 % of households in midland and lowland use bucks from the government's expected best performance. Interviews made with households during data collection show Siltie Zone livestock and fishery department tried to introduce konso buck to the lowland which was done without conducting a suitability analysis [40]. Farmers reported that introduced bucks were not desired by the community due to stunted growth and Inability to cope with the environment compared to local bucks. Furthermore, less opportunity to rear goats together with neighbors which resulted from the enclosure of communal land by the government and the changing of most of the pastureland (grazing land) to agricultural cultivation. In this case, selecting based on breeding could be the best option. Similarly, the study made by Ref. [52] shows that Genetic improvements in livestock in developing countries like Ethiopia selection based breeding schemes have the potential to provide resource-poor producers with access to improved animals that could ensure increased productivity and hence contribute to poverty reduction. Furthermore, more Weak flock separation practice was observed where the only base was young (intended for milk utilization) and diseased. Flock separation based on size, sex, productivity stage (pregnant and non-pregnant) and breeding didn't given attention which is determined as a knowledge gap.

### 4.4 Milk yield and milking length

**Fig. 1:**
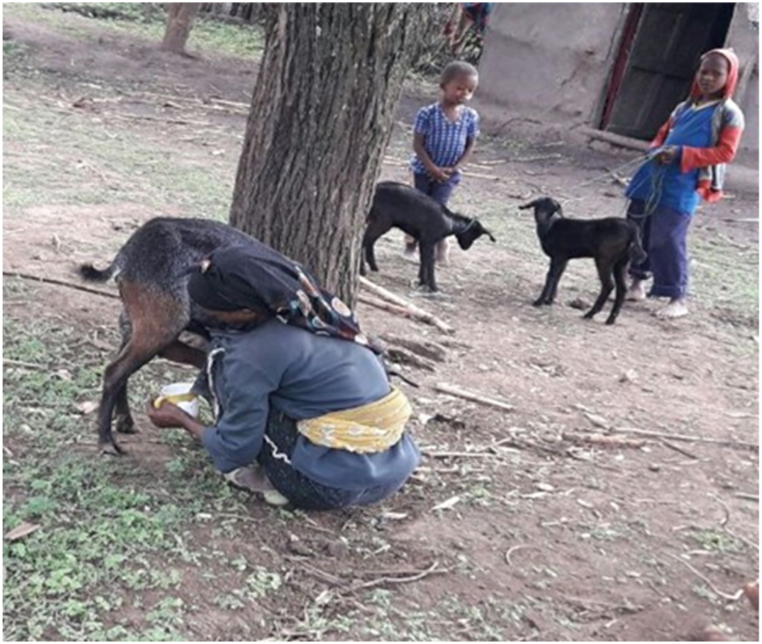
Got milking practice.

Milk yield variation (P < 0.05) was observed across agroecology where goats found at the lowland of the siltie zone recorded (0.655 ± 0.18 L) better than midland and highland. The mean value of the daily milk yield of goats reported from the current study was 0.44 ± 0.07 L/day. Comparable result of 0.4 ± 0.2 L/head/day was reported by Ref. [53] study made on Goat in Different Agro-Ecologies of Western Hararghe, and 0.422 ± 0.012 L/head/day reported by Ref. [54]Tsegaye (2011) study made on Abergelle Goat aimed at Improving Growth Rate and Milk Production Performance at Community Based Breeding Program. The daily Milk value of the current study was lower than 0.52 ± 0.03, 0.61 ± 0.02, and 0.51 ± 0.01 report of [46] who conducted a study on Arab, Felata, and Gumuz goats in North Western Low-lands of Ethiopia. The Present finding report was higher than the average daily milk yield of 0.28 kg reported by Ref. [49] a study made on Afar goats. Respondent's female goats' preference at the lowland intentioned of owning milk-yielding goats which is determined by physically observing body confirmation (body shape). Midland and highland goat selection aimed at producing the number of market goats which was based on multiple birth, goad color, and large size (see Fig. 1).

### 4.5 Milking practice of Goat at study area

**Fig. 2:**
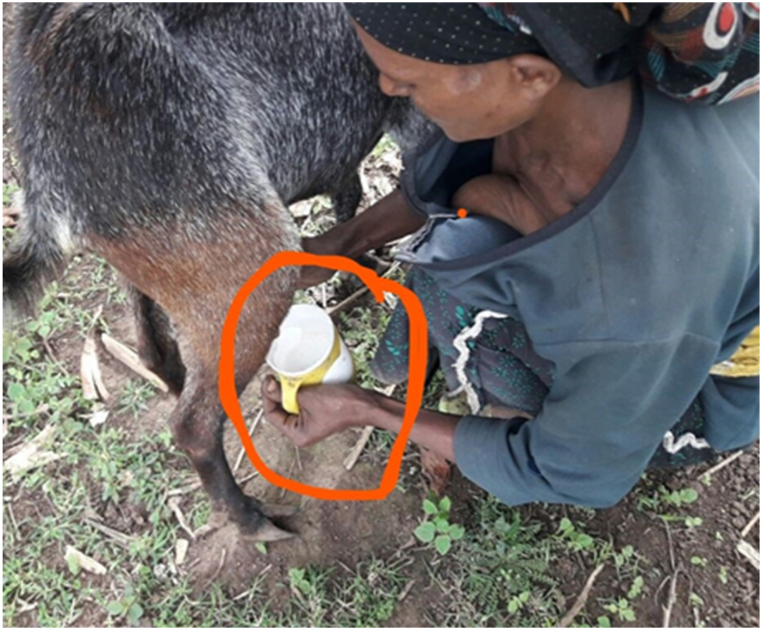
Milking equipment.

The current study showed the purpose of milking varies (P < 0.05) among agroecology. Lowland respondents milk goats majorly (85.16 %) for home consumption however, 56.25 % of midland and 75.78 % of highland respondents do not milk goats at all. In line with current findings from Lowland [21] from Tigray Tembien district reported that the majority of the milk produced from goats is used for home consumption. Moreover [2] reported that of the total annual milk production, 50 % was used for household consumption and 10 % for selling. This is because less milk yield is obtained from goats and the presence of an alternative source of milk from cattle. Plastic materials were common milking equipment utilized by 77.86 % of respondents (Fig. 2). Farmers relate the case with availability and ease of cleaning. Most of the midland and highland respondents clean milking equipment once per day however, lowland respondents clean twice per day. Lowland farmers milk goats twice per day which was in morning and night sessions while only in morning sessions at Midland and Highland. The discussion made with selected FGD infers that most of the respondents at midland and highland prefer cow milk due to milk obtained from goats being too small and left for kids to assist in growing fast.

**Fig. 3:**
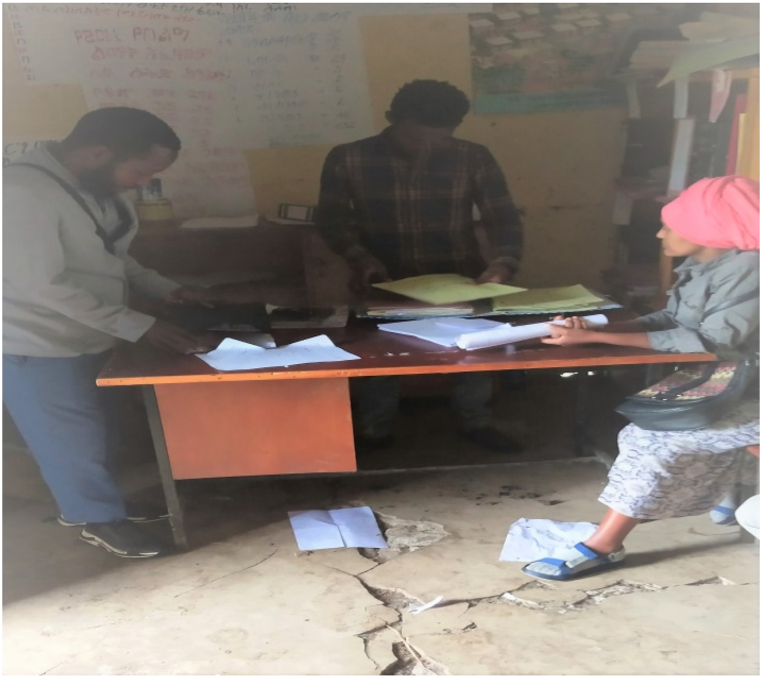
Respondents selection based on prior goat.

**Fig. 4:**
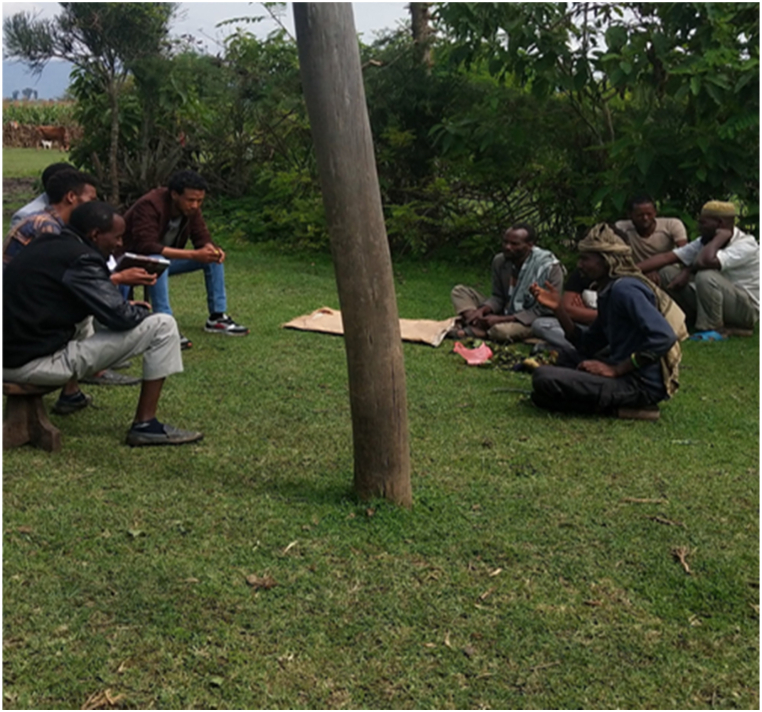
Focus Group Discussion made at kebele level rearing experience with development agents.

### 4.6 Reproductive traits performance

**Fig. 5:**
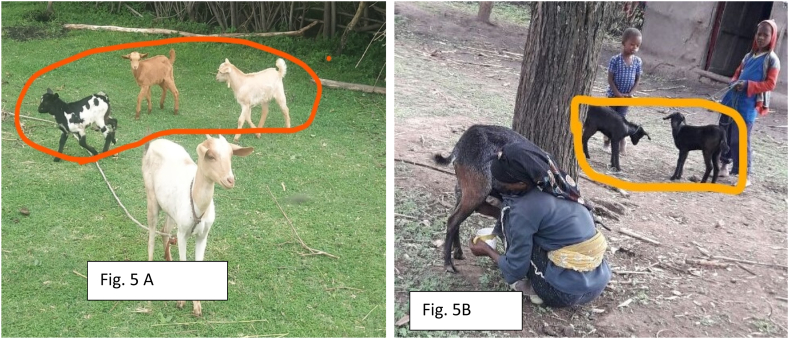
A and B Litter Size of goats from lowland study area.

Longer AFM (male), and AFS (female) which resulted in higher AFK were reported from the lowland. These characters were not considered as desirable as prolonging this resulted in a lower number of kids obtained in life. Accordingly [46] reported that higher age at first mating/service is not desirable hence; it leads to fewer numbers of kids born in a lifetime. The discussion made with selected respondents infers this is due to environmental-related cases like prolonging of the dry season resulting in feed and water shortage which in turn reduces animal performance. Moreover, a larger LS (1.95 ± 0.18) was also reported from the lowland study area. FGD from the study area revealed that higher number of LS (litter size) was observed during the rainy season compared to the dry season (Fig. 5. A and B). Farmers relate higher LS with favorable environmental conditions (sufficient feed, water availability, and favorable environmental conditions) coupled with the genetic makeup of goats. In line with this concept, favorable environmental conditions and management practices are considered important factors to affect litter size by modifying the expected value of the phenotype Apart from the genetic makeup of animals ([55,56]. LS recorded in the current study was nearly comparable to the 1.65 ± 0.08 with the report of [57] from South Gondar.

**Fig. 6:**
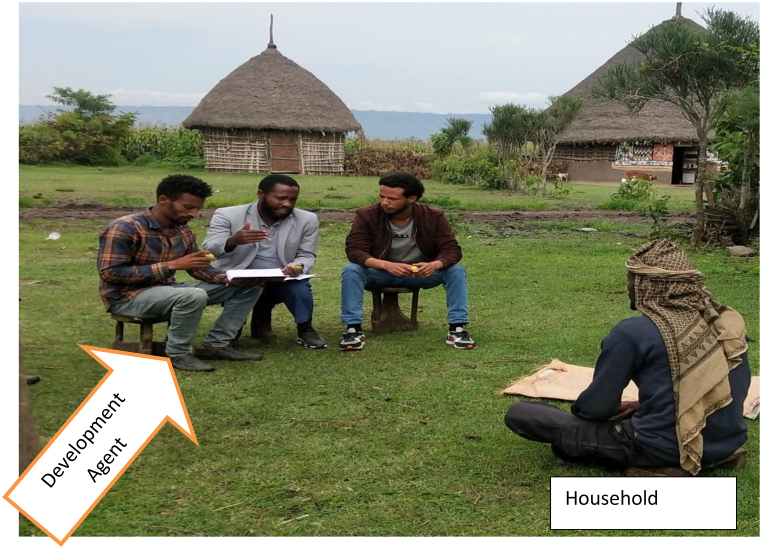
Discussion made with selected Household.

### 4.7 Selection of breeding buck and doe

This study specified Lowland respondents select breeding buck primarily by referring to body conformation, large body size, presence of horn, and behavior while breeding doe based on body conformation, udder size, milk yield, and litter size ranked 1st, 2nd, 3rd, and 4th respectively. In line [30] reported that traits like body size, body conformation (height, length, and appearance), and coat color were considered important traits and were given due emphasis in selecting breeding bucks. In accordance with this study due selection report made by different scholars ([[31], [32], [33]] revealed that body size, litter size, and milk yield were listed as preferred traits. As respondents from all study areas also reported, goats preferred for breeding, meat, and milk were selected by determining age (by looking at their teeth), presence of defects on the body (blindness, abnormal legs, and broken horn), sign of disease (presence of diarrhea) and activeness. Furthermore, the discussion made with the residents showed that they were looking for different body parts such as animal appearance (activeness, horn presence), Back side, and presence of normal teats (for females) and collect other information like mothering ability, litter size, milk yield per day during purchasing a breeding goat from the market.

### 4.8 Constraints of Goat production in study area

The identification of major constraints for a given farm animal production system in a given area is a prerequisite to plann appropriate intervention strategies [30]. The overall index from the current investigation revealed that Feed shortage (index = 0.14), water shortage (index = 0.11), and land shortage (index = 0.10) were major goat production constraints reported from the study area during the dry season. This is related to the prolonged dry season poor feed storage practices, and changing of pastureland into cropland during the rainy season. Current studies show, there were differences in constraint types across agro-ecology and seasons where, drought, water, and feed shortage at lowland; feed, water, and disease at midland and; feed and land shortage at highland ranked 1st, 2nd, and 3rd during dry season in Siltie Zone (Table 7). Feed shortage was a common primary goat production constraint in both season and agroecology. Furthermore, it was worsened by the absence of awareness and poor feed conservation techniques practice. This report agreed with [45] who stated feed shortage was the major problem faced by goat keepers. Moreover [37], reported that feed shortage was a limiting constraint in both dry and wet seasons.

## 5 Conclusion and recommendation

### 5.1 Conclusion

In this study goat Breeding and Milking practices and Goat Production Constraints were assessed under three agroecology of the Siltie Zone. A multistage sampling technique was followed and 384 respondents were selected and information was collected through questionnaires, FGD, and field observation. Role differences were observed among genders where males were fixed to outdoor activities and females in and or around the home. Farmers handle goats for multiple purposes, in which income, milk, meat, and breeding were prioritized by respondents. Most of the breeding bucks originated from respondents' flocks due to less chance of improved breed and communal land enclosure by the government for rehabilitation purposes. Weak flock separation ideology and practice were determined as a major focus were disease followed by age (only young in lowland) beyond which inbreeding, productivity (pregnant) and size matters are given little or no attention. Lowland goats were comparably potential milk producers (yielders) and multiple births (LS) mainly during the rainy season. This shows Favorable environmental condition (sufficient feed, water availability, and favorable environmental condition) coupled with the genetic makeup of goats supports the efficiency of larger LS. Most highland and midland farmers do not milk goats for consumption or sale due to less milk yield and more do they depend on cow milk. Feed shortage was a common goat production constraint at both seasons and in all agroecology which resulted from the prolonged dry season, enclosure of communal land by the government at lowlands, and cultivation of most of the land during the rainy season under all agroecology.

### 5.2 Recommendation

Up on findings from this study, the following recombination was forwarded by researchers:

✓ Characterization of an indigenous goat at Siltie zone should be conducted to prepare suitable ground for future breeding based improvement activities
✓ Farmers should be trained on flock separation, goat milk importance (midland and highland farmers), and feed storage should be tackled by creating an opportunity to access Alternative feed sources like improved forage cultivation, hay, and crop-by-product
✓ Improvement through selection should be implemented for milk potential and litter size at the community level to come up with an environmentally sustainable and productive goat breed.

## CRediT authorship contribution statement

**Ahmed Hussein:** Writing – review & editing, Writing – original draft, Methodology, Formal analysis, Data curation, Conceptualization. **Serkalem Aseffa:** Writing – review & editing, Validation, Resources. **Belete Kuraz:** Visualization, Supervision, Resources. **Bushra Bedaso:** Visualization, Supervision.

## Declaration of competing interest

The authors declare that they have no known competing financial interests or personal relationships that could have appeared to influence the work reported in this paper.
