# Supplementary material for: Breeding and milking managements and Goat production constraints in Siltie Zone SNNPR, Ethiopia
Source: Heliyon. 2023 Nov 22;9(12):e22573. doi: 10.1016/j.heliyon.2023.e22573 (PMC10724559; doi:10.1016/j.heliyon.2023.e22573)
Supplement: Multimedia component 1 [file mmc1.doc]

# Appendix

# Appendix1. Questionnaire used for the surveys

Selection Practices, Milking Practice, Reproductive Performance and Constraints of Goats Production at household level is a plan of this proposal to conduct study at selected study area of silte Zone.

**Messages for the data collector:** dear Data collector before starting the data collection, be sure their local language, Traditional Respecting of an area, choose places suitable for respondents and for observation of local goats. Identify proper time which is suitable for respondent before beginning data collection. While filling the questionnaire ask the respondent politely by respecting, listen carefully and write correctly the answer. If the answer is not clear, describe the question again by providing example of locally available materials by politely. Lastly be sure all question has been fully addressed and filled correctly.

**Part one: - General Information**

**I. Data compilation** Questionnaire No: -----------------

Name of the enumerator___________________________________. Date ______/______/2019

Agro-ecology ___________________. Woreda______________________. kebele ___________

**II. Household Information**

**A. Demography, Profession and Education level**

1. Names of Respondents_________________________________. Age____________

2. Sex: 1. Female
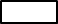
 2. Male
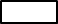


3. Marital Status**:** 1. Single 2. Married 3. Widowed 4. Divorced

**4. Occupations:**

1. Farmer 2. Self-employed 3. Employee. 4. Student. 5. Unemployed . 6.1 and 2. 7. 1and 3 8. 2 and 3 9. other

5. Education level: 1. Illiterate
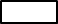
 2.traditional/church education
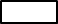
 3.Grade 1-8
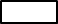
 4.Grade 9-12
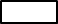
 5.Certificate
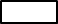
 6.Diploma
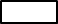
 7.Degree
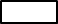
 8. Others
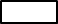


**I. purpose handling Goat**

1. What are the purposes of keeping goats and rank your answer?

| S/n | Purposes of rearing goats | index | Rank |
| --- | --- | --- | --- |
| 1 | For meat |  |  |
| 2 | For milk |  |  |
| 3 | income |  |  |
| 4 | breeding |  |  |
| 5 | saving |  |  |

**II. Labor management at household level**

**Table 2. Labor Management for Different Livestock Related Activities under Study Area**

| Activities | Agro ecology | Sons (%) | Daughter (%) | Father (%) | Mother (%) | Hired labor (%) | P Value |
| --- | --- | --- | --- | --- | --- | --- | --- |
| Herding of large ruminant (cow, ox, heifer and bull) | Lowland |  |  |  |  |  |  |
|  | Midland |  |  |  |  |  |  |
|  | Highland |  |  |  |  |  |  |
|  | Overall |  |  |  |  |  |  |
| Herding of small ruminant (sheep and goat) | Lowland |  |  |  |  |  |  |
|  | Midland |  |  |  |  |  |  |
|  | Highland |  |  |  |  |  |  |
|  | Overall |  |  |  |  |  |  |
| Caring of young, sick animals and | Lowland |  |  |  |  |  |  |
|  | Midland |  |  |  |  |  |  |
|  | Highland |  |  |  |  |  |  |
|  | Overall |  |  |  |  |  |  |
| Milking animals (goats) | Lowland |  |  |  |  |  |  |
|  | Midland |  |  |  |  |  |  |
|  | Highland |  |  |  |  |  |  |
|  | Overall |  |  |  |  |  |  |
| Cleaning of house and barn | Lowland |  |  |  |  |  |  |
|  | Midland |  |  |  |  |  |  |
|  | Highland |  |  |  |  |  |  |
|  | Overall |  |  |  |  |  |  |
| Buying and selling of live animals | Lowland |  |  |  |  |  |  |
|  | Midland |  |  |  |  |  |  |
|  | Highland |  |  |  |  |  |  |
|  | Overall |  |  |  |  |  |  |
| Selling of animal products (milk, cheese, butter and yoghurts) | Lowland |  |  |  |  |  |  |
|  | Midland |  |  |  |  |  |  |
|  | Highland |  |  |  |  |  |  |
|  | Overall |  |  |  |  |  |  |
| Building human house and livestock shelter | Lowland |  |  |  |  |  |  |
|  | Midland |  |  |  |  |  |  |
|  | Highland |  |  |  |  |  |  |
|  | Overall |  |  |  |  |  |  |

**IV. Breeding practices
Table 3. Breeding practices of goat at study area**

| Variables | | under three agro-ecology |
| --- | --- | --- |
|  |  | N (%) |
| Breeding Buck Source | Own flock |  |
|  | Neighbors |  |
|  | Government |  |
| Do You Separation Flock | Yes |  |
|  | No |  |
| Basis for flock separation | Ages |  |
|  | By Sex |  |
|  | Condition |  |
| know and prevent Inbreeding problem | Yes |  |
|  | No |  |

IV. Milk Yield and Lactation Length

Table 4: Average percentage of Productive performance of goats at study area

| Variables | Lowland | Midland | Highland | Overall |
| --- | --- | --- | --- | --- |
|  |  |  |  |
| Milk yield (Litter ) |  |  |  |  |
| Lactation length (Month) |  |  |  |  |

V. Milking practice at household level

**Table 5: Milking Practice at Study Area**

| Activities | | under three agro-ecology | |
| --- | --- | --- | --- |
|  |  | N | % |
| Purpose of milking | Home consumption |  |  |
|  | Income (sale) |  |  |
|  | Not milked |  |  |
| Milker | Mother |  |  |
|  | Young female |  |  |
| Milk utilizers | Children |  |  |
|  | Elders |  |  |
|  | Women |  |  |
| Milking equipment | Plastic |  |  |
|  | Metallic |  |  |
|  | Traditional |  |  |
| Frequency of cleaning | Once per day |  |  |
|  | Twice per day |  |  |
| Milk cleaning material | Water and soap |  |  |
|  | Water and fumigation |  |  |
|  | Water, soap and fumigation |  |  |

**VI. Reproductive Performance of goats from study area**

**Table 6. Reproductive Performance of goats from study area**

| Reproductive Variables | under three agro-ecology (N) |
| --- | --- |
| AFM (month) |  |
| AFS (month) |  |
| AFK (month) |  |
| KI (month) |  |
| LS (number) |  |

**VII. Goat selection practice by household**

Table7. Index and rank Goat selection criteria and practices in study area

| **Goat selection criteria** | under three agro-ecology | | | |
| --- | --- | --- | --- | --- |
|  | Male | | Female | |
|  | Index | Rank | Index | Rank |
| Coat color |  |  |  |  |
| Behavior |  |  |  |  |
| Large Body size |  |  |  |  |
| Presence of horns |  |  |  |  |
| Udder sizes |  |  |  |  |
| Litter sizes |  |  |  |  |
| Body conformation |  |  |  |  |
| High Milk yield |  |  |  |  |
| Disease tolerance |  |  |  |  |

**VIII. Goat production Constraints**

**Table 8: index of goat production Constraints of Goats production in study area**

| Variables | Lowland | | Midland | | Highland | | Overall | | | |
| --- | --- | --- | --- | --- | --- | --- | --- | --- | --- | --- |
|  | Dry | Rainy | Dry | Rainy | Dry | Rainy | Dry | | Rainy | |
|  | index | index | index | index | index | index | index | Rank | index | Rank |
| Drought Problem |  |  |  |  |  |  |  |  |  |  |
| Water Shortage |  |  |  |  |  |  |  |  |  |  |
| Feed Shortage |  |  |  |  |  |  |  |  |  |  |
| Land Shortage |  |  |  |  |  |  |  |  |  |  |
| Disease and Parasite |  |  |  |  |  |  |  |  |  |  |
| Health Service |  |  |  |  |  |  |  |  |  |  |
| Productive Breeds |  |  |  |  |  |  |  |  |  |  |
